# A Little Goes a Long Way: Low Working Memory Load Is Associated with Optimal Distractor Inhibition and Increased Vagal Control under Anxiety

**DOI:** 10.3389/fnhum.2017.00043

**Published:** 2017-02-03

**Authors:** Derek P. Spangler, Bruce H. Friedman

**Affiliations:** Department of Psychology, Virginia Polytechnic Institute and State University, BlacksburgVA, USA

**Keywords:** anxiety, heart rate variability (HRV), distractor interference, working memory, inhibition

## Abstract

Anxiety impairs both inhibition of distraction and attentional focus. It is unclear whether these impairments are reduced or exacerbated when loading working memory with non-affective information. Cardiac vagal control has been related to top–down regulation of anxiety; therefore, vagal control may reflect load-related inhibition of distraction under anxiety. The present study examined whether: (1) the enhancing and impairing effects of load on inhibition exist together in a non-linear function, (2) there is a similar association between inhibition and concurrent vagal control under anxiety. During anxiogenic threat-of-noise, 116 subjects maintained a digit series of varying lengths (0, 2, 4, and 6 digits) while completing a visual flanker task. The task was broken into four blocks, with a baseline period preceding each. Electrocardiography was acquired throughout to quantify vagal control as high-frequency heart rate variability (HRV). There were significant quadratic relations of working memory load to flanker performance and to HRV, but no associations between HRV and performance. Results indicate that low load was associated with relatively better inhibition and increased HRV. These findings suggest that attentional performance under anxiety depends on the availability of working memory resources, which might be reflected by vagal control. These results have implications for treating anxiety disorders, in which regulation of anxiety can be optimized for attentional focus.

## Anxiety, Inhibition, and the Enhancing Effects of Load

### Anxiety Impairs Inhibition

Anxiety and other negative states tend to grab attention and disrupt the ability to inhibit irrelevant stimuli, even when those stimuli have little affective quality (e.g., Pallak et al., 1975; Hart et al., 2010; Dolcos et al., 2011; Choi et al., 2012). This impairment of inhibition might be attributed to cognitive aspects of anxiety (e.g., worry; Mathews, 1990; Borkovec et al., 1998). By consuming limited WM resources, anxiety reduces the capacity for distractor inhibition (Eysenck and Calvo, 1992; Hayes et al., 2008; Verkuil et al., 2009; Lavie, 2010).

### A Potential Solution: Working Memory as a Core Feature of Anxiety Regulation

Persistent anxiety and its attentional deficits are often treated with interventions that target the enhancement of emotion regulation (ER) skills that rely on WM (e.g., cognitive restructuring; Beck, 1979; Hofmann and Asmundson, 2008; Hofmann et al., 2009). A shared feature of many cognitive ER strategies is WM load, which involves filling up the capacity-limited “blackboard” for conscious thought (i.e., WM) with non-affective material (e.g., a letter string; Baddeley, 1992; Engle, 2002; Ochsner and Gross, 2008; van Dillen et al., 2009; Buhle et al., 2014). Therefore, in the current study, WM load is conceptualized as a core mechanism underlying voluntary, top–down regulation of emotion and anxiety. WM load increases tend to reduce anxiety and other negative emotional states by shifting cognitive resources away from emotion-laden thoughts (e.g., worry; van Dillen and Koole, 2007; van Dillen et al., 2009; Kanske et al., 2011; King and Schaefer, 2011). Through attenuating anxiety in this way, WM load increases also reduce anxiety-related impairments to inhibition and selective attention (Schutz and Davis, 2000; Bradley et al., 2010; van Dillen and Derks, 2012; Vytal et al., 2012; Clarke and Johnstone, 2013).

### Cardiac Vagal Control Relates to Performance-Enhancing WM Load

The notion that WM load enhances concurrent inhibition is consistent with the *Neurovisceral Integration Model* (Thayer and Lane, 2000, 2002, 2009). In this view, prefrontal cortex (PFC) areas related to WM tonically suppress subcortical areas important for anxiety and worry. Such PFC-mediated suppression is manifested as augmented cardiac vagal control, the vagus nerve’s inhibitory effect on heart rate (HR; Berntson, 1997; Ter Horst and Postema, 1997). Cardiac vagal control is often quantified by high-frequency variability in the HR time series that often occur in phase with oscillations in respiration (HF-HRV; Malliani et al., 1991). HRV will be hereinafter used to refer to vagally mediated HF-HRV. High HRV at rest and during tasks has been speculated to proxy PFC-mediated cognitive regulation (perhaps load-dependent regulation; see below) of negative emotional states, including anxiety (for reviews, see Thayer and Lane, 2002; Appelhans and Luecken, 2006; Friedman, 2007). High cardiac vagal control (i.e., high HRV), through reflecting the degree of cognitive regulation over performance-harming anxiety or “stress,” has been linked to improved inhibition and attentional performance (e.g., Hansen et al., 2003; Johnsen et al., 2003; Thayer et al., 2009; Elliot et al., 2011).

In the present study, we focused on HRV responses that relate to state regulatory efforts, as opposed to resting HRV, which reflects trait processes (Thayer et al., 2012). Within-subject increases in HRV might relate to WM load that regulates anxiety and enhances inhibition (Thayer and Lane, 2009). This possibility is supported by a number of studies. First, within-person increases in HRV tend to co-vary with ER strategies (e.g., reappraisal and expressive suppression) that load WM (Butler et al., 2006; Denson et al., 2011). Second, high HRV has been associated with simultaneously heightened dorsolateral PFC (a WM-related brain area) activity that relates to both reduced emotionality and increased WM load (Lane et al., 2009; Qin et al., 2009).

## Anxiety, Inhibition, and the Impairing Effects of WM Load

### WM-Dependent ER and HRV as Costs to Inhibition

Contrary to the above-cited research, engagement in WM-dependent ER can impair performance on concurrent or subsequent tasks that require inhibition (Friese et al., 2013; Ortner et al., 2013). These effects may be explained by the *Load Theory of Selective Attention and Cognitive Control*, in which WM capacity is required for inhibition of distractor interference (de Fockert et al., 2001; Lavie et al., 2004). In this sense, ER’s inherent WM load is thought to reduce WM capacity for maintaining inhibition-related goals. As an indicator of WM-dependent ER, task levels of HRV might relate to ongoing impairments to inhibition driven by usage of WM resources. Partially supporting this notion, subjects with relatively higher resting (but not task) HRV showed a greater likelihood to use WM-dependent ER during a negative emotion picture paradigm, but showed worse performance on a subsequent Stroop task (Pu et al., 2010). In this prior study, it is possible that high HRV was associated with impaired inhibition because individuals with high HRV exhausted WM resources during ER.

## Non-Linear Model of WM Load and Inhibition Under Anxiety

**FIGURE 1:**
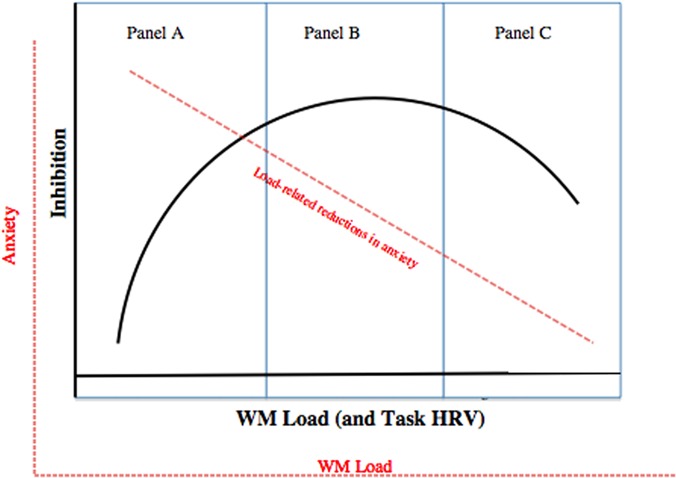
**Hypothesized quadratic association between WM load and inhibition under high state anxiety.** Sub-axes with dotted lines reflect association between concurrent anxiety levels and inhibition. **(A)** Represents no load (0 digits) followed by low load (2 digits). **(B)** Reflects moderate load (4 digits), and **(C)** represents high load (6 digits).

Evidence for the deleterious impacts of WM load and of HRV on concurrent inhibition during anxiety is perplexing, in view of work that highlights the performance-enhancing qualities of WM load. Rather than treating these differing results as incompatible, it may be that both negative and positive relations among WM load and inhibition exist together within a larger non-linear function (Marcovitch et al., 2010). A novel theoretical model is presented here that specifies a quadratic relation between inhibition and WM load under high anxiety, with WM load being conceptualized as a core mechanism of anxiety regulation (Hendricks and Buchanan, 2015; **Figure 1**). In this quadratic function, increased load may help inhibition by reducing anxious cognitions when such increases are in the range of no load to moderate load (i.e., when minimal WM resources are drained from the concurrent inhibition task; **Figure 1A**). In parallel, WM load increases are also hypothesized to deplete shared resources, which may counteract any performance-enhancing effects, thereby flattening the positive load-inhibition relation from no to moderate load (**Figure 1A**). Moderate load may represent a critical point past which too much WM capacity is used; additional load impairs the ability to reduce distractor interference and hence causes an increasingly negative load-inhibition association (Lavie, 2005; Berggren et al., 2013; Ortner et al., 2013; **Figure 1C**). In that task HRV levels have been speculated to represent a bodily manifestation of load-related regulation of anxiety (i.e., WM load), a nearly identical quadratic function between HRV and concurrent inhibition was predicted (see **Figure 1**; Lane et al., 2009).

Partial support for the theoretical model came from a study that showed a quadratic HRV-performance relation in individuals who frequently use a WM-dependent ER strategy (i.e., *expressive suppression*; Spangler et al., 2015). Because cognitive resources were not manipulated, these effects may be attributable to other factors than WM, such as moderate HRV reflecting optimal levels of arousal for performance (see Marcovitch et al., 2010 for a similar quadratic function in children).

## Current Study

The primary aim of the current study was to examine quadratic associations between WM load and task HRV to inhibition under high state anxiety, in order to test whether intermediary levels of WM load and vagal control optimize distraction inhibition during anxiety. These aims were approached in an experiment that combined an anticipatory noise blast paradigm (to induce anxious cognition) with a common dual WM-inhibition task adapted from Lavie et al. (2004). While being either safe from or under threat of noise (Patrick and Berthot, 1995; Grillon et al., 2008, 2009), subjects loaded WM capacity and simultaneously completed an Eriksen flanker task, a common measure of inhibition (Lavie et al., 2004).

Under situations of high state anxiety (threat trials), WM load was predicted to show a negative quadratic association with inhibition performance (Hypothesis 1). An exploratory corollary of this hypothesis was that the function’s shape would resemble that depicted in **Figure 1**, such that moderate load would be associated with relatively optimal inhibition. Due to its theoretical links with WM-dependent regulation of anxiety (Lane et al., 2009), task HRV was predicted to have a positive linear relation with WM load under high state anxiety (threat trials; Hypothesis 2**).** Insofar that WM load is related to HRV during high anxiety, we predicted that task HRV would also show a negative quadratic association with inhibition performance (Hypothesis 3).

Special focus was given to contrasting relations of WM load and HRV to inhibition between threat and safe trials, in order to investigate unique mechanisms accounting for the “threat” function. Load increases under low anxiety (i.e., safety) should drain WM capacity without performance enhancements via anxiety reduction. Therefore, in testing relations of inhibition to load and HRV under safety, a general absence of curvilinearity was predicted. Hypotheses were tested with a series of multilevel models that were conducted with and without self-reported subjective anxiety as a covariate, in order to assess whether the resultant functions were driven by emotional factors rather than by WM load.

## Materials and Methods

### Subjects

Subjects were 120 (68 female) undergraduates at Virginia Tech (*M*_age_ = 19.3 years, *SD*= 2.8 years), who were recruited both online and with flyers posted on campus. Participation was compensated with extra credit in a psychology course. Exclusionary criteria were made on the basis of self-reported: (1) cigarette smoking or tobacco use, (2) diagnoses of cardiovascular disease, (3) and psychiatric/neurological disorders. Subjects were instructed to abstain from alcohol for 24 h, caffeine for 12 h, food for 2 h, and vigorous exercise for 2 h prior to participation. Four subjects out of 120 enrolled were excluded due to equipment malfunction, yielding 116 subjects retained for analyses (66 female; *M*_age_ = 19.1 years, *SD* = 1.85 years). This study was approved by the Virginia Tech Institutional Review Board, and informed consent was obtained from all subjects.

### Procedure

Subjects were greeted by the experimenter upon arrival at the lab and informed of the nature of the study and the noise blast paradigm. After providing written consent, subjects were attached to physiological recording equipment, and they completed self-report questionnaires. Next, two practice trials of the experimental task were conducted with noise delivery, and subjects were given the opportunity to ask questions about the task.

After the first physiological baseline recording, subjects performed the experimental task, which was comprised of 28 trials, of which there were seven trials for each level of WM load (0, 2, 4, 6; see below). Each trial included a series of flanker responses (to measure inhibition) as well as WM maintenance; a typical trial is described in detail under the Experimental Task section below. Twelve of the 28 trials involved safety from noise blast (three safety trials per level of load), while another 12 trials included threat of noise blast without actual noise delivery (three threat trials per load level). Of importance, an additional four trials included threat of noise blast with the delivery of actual noise (1 blast trial per level of load). This design yielded 24 retained trials (12 trials for safety and 12 trials for threat); the four threat trials with noise blast were excluded from data analysis due to the confounding effects of startle and pain on performance (Kalisch et al., 2006). In threat trials, delivery of noise, or lack thereof, was randomized with the qualification that 25% of threat trials would involve actual noise (see Procedure above; Kalisch et al., 2006). In these trials, the timing of the blasts was randomly determined so that only one noise occurred during the flanker/WM section of the trial. Randomization of trial and stimulus delivery was implemented within the DMDX software (Forster and Forster, 2003).

Each subject completed all 28 trials and thus experienced 4 threat trials and 3 safety trials for every WM level (0, 2, 4, and 6). For the WM manipulation, digit series lengths of 0, 2, 4, and 6 were chosen to correspond to no, low, moderate, and high WM load, respectively (Lavie et al., 2004; van Dillen et al., 2013). To avoid switching costs, the seven trials with the same level of WM load were blocked together, and safety/threat was randomly counterbalanced within each of the WM blocks (Lavie et al., 2004). This created four WM load blocks that were randomly counterbalanced. Each of the four WM blocks was preceded by a 3-min “vanilla” baseline that was composed of a calming nature film (Jennings et al., 1992). Multiple baselines were incorporated for a more accurate representation of Task HRV for each level of WM. The entire run of the experiment lasted about 1 h.

### Experimental Task

**FIGURE 2:**
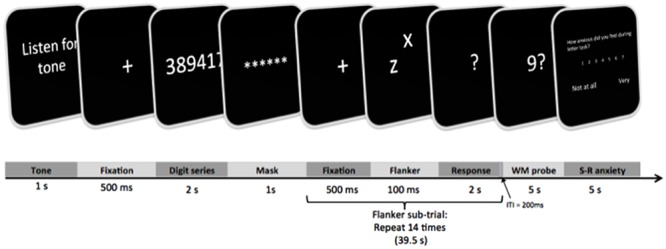
**Example of a trial from the experimental task**.

A typical trial of the experimental task is depicted in **Figure 2**. Trials were scripted and presented on a PC using DMDX software (Forster and Forster, 2003). At the beginning of the trial, subjects heard one of two tones via headphones. A low tone indicated safety (0% chance) from white noise blast, and a high tone indicated threat (i.e., “some possibility”) of noise blast that may be delivered at some point in the upcoming trial. Following tone presentation, a fixation cross appeared on the computer screen for 500 ms. Next, subjects saw a series of digits that were presented for 2 s. When subjects viewed these numbers, they were expected to silently keep in mind the digits for the remainder of the trial, rather than focus on their emotions. They were also expected to correctly answer a recognition probe later in the trial. The length of the presented series (# digits) varied between 0, 2, 4, and 6, depending on the WM block. For each trial, digits in the series were chosen at random from 1 to 9 with the following qualifications: no more than two consecutive digits could appear in the series (e.g., 1, 2, 3, 7), and the same numbers could not be used twice (e.g., 7, 7, 9, 5; Lavie et al., 2004). Once the series disappeared, subjects rehearsed the digit series for the remainder of the trial. Specifications for digit presentation were adapted from Lavie et al. (2004), with the added instructions that subjects should maintain the series rather than focusing on their emotions. A masking array was subsequently presented for 1 s to visually orient subjects for the upcoming flanker task.

While maintaining digits silently, the subject completed a series of 14 flanker sub-trials for each of the 28 trials of the experimental task. Each flanker sub-trial began with a 500 ms fixation cross. In accord with Lavie et al. (2004), a single flanker sub-trial consisted of a target letter in lowercase (the letters *x* or *z*) that was presented in the middle of the screen. At the same time, a peripheral distractor letter was presented at a subtended location relative to the target. For each response, subjects were asked to ignore the peripheral letter and to classify the target letter as an *x* or *z* by typing 1 or 2, respectively, on a computer keyboard. The visual array of letters lasted 100 ms, and then subjects were given 2 s to classify the letter with a typed response. Length of sub-trials did not vary by subjects’ response times, such that the next flanker sub-trial was always initiated after 2 s. The intertrial interval for flanker sub-trials was 200 ms. The flanker sub-trials differed such that the target was either congruent with the peripheral letter (e.g., target *z* and peripheral *z*) or incongruent with the peripheral letter (e.g., target *z* and peripheral *x*, or target *x* and peripheral *z*). In each overall trial (safe or threat), 7 of the flanker sub-trials were incongruent and the other 7 sub-trials were congruent, with their order being randomized within each of the 28 trials.

Unlike other studies, 14 sub-trials were combined to create a ∼39.5 s period of dual task performance (flanker responses with concomitant WM maintenance). This was done to satisfy the 30 s length as a potential minimum for reliable HRV recording (G. Berntson, personal communication, September 10, 2014; Task Force of the European Society of Cardiology and the North American Society of Pacing and Electrophysiology, 1996).

After the series of flanker sub-trials with WM maintenance, a recognition probe appeared on the screen for 5 s. The probe consisted of a single letter; the subject indicated whether or not the letter appeared in the preceding series by pressing 1 or 2, respectively. At the end of the trial, subjects were given another 5 s to retrospectively report anxiety experienced during the preceding flanker/WM task. A single trial of the experimental task lasted approximately 55 s, and the task was scripted so that the length of trials did not vary by response time.

### Measures and Apparatus

#### Experimental Task Measures

##### WM Load

Digit series length during the flanker task varied such that load level assumed the following values for each subject: 0, 2, 4, and 6. Although these values were the result of an experimental manipulation (see above), they are conceptualized as a variable that is on a continuous ratio-like scale (Braver et al., 1997).

##### Flanker Performance

Response times (RT) to classify target letters amidst distractors were collected. Accuracy in classifying letters was also attained, but RT was of primary interest for hypothesis testing. As in Lavie et al. (2004), only RTs from correct classifications were included in interference calculations and analyses. Interference scores were computed by subtracting RTs of congruent trials from that of incongruent trials, and this difference score served as a measure of inhibition performance (Stins et al., 2004; Dennis and Chen, 2007; Hart et al., 2010; Qi et al., 2014). This method yielded 28 interference scores for each person (one difference score for each of the seven threat and seven safe trials for each level of WM load). To better compare results to that of other studies, interference scores were reverse scored before being entered into data analyses, such that higher levels on this measure indexed relatively better inhibition. Higher inhibition scores reflected smaller differences between congruent and incongruent trials in RTs to classify targets, suggesting relatively better suppression of incongruent stimuli (Lavie et al., 2004).

##### Subjective Anxiety

At the end of each trial (threat, safe), anxious experience was reported on a 7-point Likert Scale to the following question: “How anxious did you feel during the letter task?” Higher numbers indicated greater state anxiety, such that 1 indicated that anxiety was “not at all” present and 7 denoted that anxiety was “very” present (van Dillen et al., 2009).

#### Self-Report Questionnaires

##### Health History

Information was collected about health issues that could potentially confound the validity of study findings. This questionnaire allowed experimenters to validate whether subjects followed the abstention recommendations outlined above.

#### Physiological Measures

Electrocardiography (ECG) was continuously recorded throughout the experimental session. ECG was collected with Ag/AgCl spot electrodes on the subject’s thorax at a modified Lead II configuration in which one electrode at the right collarbone and the other at the bottom left rib. Analog ECG was amplified with the ECG100C (Biopac Systems Inc., Goleta, CA, USA), and then integrated and sampled with an MP150 device (Biopac Systems Inc., Goleta, CA, USA). Digital signals were next routed and saved to a PC in the next room for oﬄine analysis using AcqKnowledge software (Version 4.3). A modified Pan-Tompkins algorithm was conducted on ECG waveforms to identify R-spikes. R-spikes that were missing or misclassified due to motion artifact (which occurred in less than <1% of R-spikes) were manually identified and corrected in the ECG record. The rare cases of unidentifiable and ectopic beats (<1% of R-spikes) were removed from the ECG time series (Lippman et al., 1994). Interbeat intervals (IBI) were then computed from the ECG signal as the distance between consecutive R-spikes in millisecond (ms) units. Using Kubios software (Version 2.2), HRV was derived from the IBI signal using a Fast Fourier Transform function and quantified as spectral power (ms^2^) in the domain of normal respiration (0.15–0.4 Hz; Task Force of the European Society of Cardiology and the North American Society of Pacing and Electrophysiology, 1996).

Separate HRV estimates were yielded for all four baseline periods. Baseline HRV was derived from the last 2 min of each 3-min IBI time series in order to remove vagal influences related to cardiac stress recovery. To assess task-specific HRV accounting for baseline levels, a series of reactivity difference scores was computed for each trial by subtracting HRV during flanker performance/WM maintenance (duration = 39.5 s) from the preceding baseline HRV value (Llabre et al., 1991). This process yielded 28 different task HRV values per subject. Prior to creating differences scores, HRV values were log transformed to normalize their distribution. Task IBI levels were calculated with reactivity difference scores in the same manner.

##### Noise Blast Apparatus

The aversive stimulus was a 3-s, 105 dB blast of PC-generated white noise (adapted from Grillon et al., 2008, 2009). Noise level was controlled with an external amplifier, and noise blasts were delivered via headphones.

### Data Analyses

Variables were inspected for skew and both HRV and self-reported anxiety were log transformed to normalize their distributions. Before entering data into analyses, severe outliers were excluded (>3.5 SD). With this criterion, 18 inhibition scores (out of 2,784 scores across participants; <1%) and 11 Task HRV values (out of 2,784 values; <1%) were excluded. Hypotheses were tested with a series of random intercept models, a type of multilevel model that accounts for nesting of observations (inhibition performance, HRV) within subjects with a random slope (Raudenbush and Bryk, 2002). This method, unlike ordinary least squares regression, prevents violating assumptions of non-independence and allows for more fine-grained estimation of within- (Level-1) and between-subject (Level-2) variation (Kreft and de Leeuw, 1998). Level-1 intercepts were allowed to randomly vary between subjects and all other predictors were estimated as fixed effects, and can be interpreted as the average of within-person relations among inhibition, HRV, and load across subjects (Kreft and de Leeuw, 1998). The structure of primary models are specified below.

Working memory Load and its quadratic effect were treated as continuous variables. Quadratic terms for WM Load were built by first mean centering and then squaring WM Load’s linear term (Cohen et al., 2003). Linear and curvilinear by linear interactions between WM Load and other variables (e.g., Trial and Trait Anxiety) were computed by multiplying terms, and these interactions were probed with simple slope analysis (Aiken and West, 1991; Cohen et al., 2003). In each model, Level-1 continuous variables (e.g., WM Load, Self-reported Anxiety, HRV) were group-mean centered to reduce multicollinearity and to aid interpretation of coefficients (Kreft et al., 1995). For each hypothesis, analyses were conducted to substantiate significant interactions of load or HRV with Trial (Threat, Safe) before testing hypothesized relations in threat and safety contexts separately (Robinson et al., 2013). Trial (Threat, Safe) was coded as a dummy variable, such that 0 and 1 represented threat and safe trials, respectively.

All multilevel models presented in relation to primary hypotheses included self-reported anxiety as a covariate. This was done because, counter to the theoretical model, results indicate that WM load increases were met with increases rather than decreases in anxiety (see below). As such, it became increasingly desirable to examine relatively “pure” effects of WM load and their physiological correlates (Task HRV) apart from the unexpected changes in anxiety.

#### Multilevel Models for Hypotheses

To test Hypothesis 1, a comprehensive random intercept model was used to assess whether the quadratic association between WM load and performance was moderated by Trial (Safe, Threat).

Level-1: Flanker performance = β_0j_ + β_1j_ (WM Load)_ij_ + β_2j_ (WM Load)^2^_ij_ + β_3j_ (Trial)_ij_ + β_4j_ (Self-reported anxiety)_ij_ + β_5j_ (WM Load × Trial)_ij_ + β_6j_ (WM Load^2^× Trial)_ij_ + R_ij_

Level-2: β = ϒ_00_ + U_0j_

If the interaction between WM Load^2^ and Trial was significant, models containing WM Load, WM Load^2,^ and self-reported anxiety as predictors and flanker performance as the outcome measure were conducted for threat and safe trials separately (Cohen et al., 2003; Robinson et al., 2013).

Hypothesis 2 was tested with random intercept models of the same form as that which was used to examine Hypothesis 1, except flanker performance was replaced by Task HRV as the outcome measure. Quadratic terms for WM Load were modeled for exploratory purposes. To test Hypothesis 3, models were conducted that were identical to those used for Hypothesis 1, except that linear and quadratic terms for WM Load were switched for Task HRV and Task HRV^2^, respectively. Analyses relating to manipulation checks and basic model tenets were conducted with a series multilevel models and t-tests.

## Results

### Manipulation Checks

#### Anxiety Manipulation on Self-Report

The effectiveness of the anticipatory noise blast paradigm in increasing state anxiety was examined with a random intercept model, in which Trial (threat, safe) was modeled as a fixed effect on trials from the 0 Load condition (i.e., when there were little to no WM demands). This analysis generated a significant effect of Trial (*B* = -0.690, *p* < 0.001), which suggests that during no WM load there were higher levels of subjective anxiety during threat than in safe trials. Descriptive statistics for all variables appear in **Table 1**.

**Table 1:** Means (standard deviations) of performance, cardiac, and self-report measures.

	Threat trials				Safe trials			
	Load 0	Load 2	Load 4	Load 6	Load 0	Load 2	Load 4	Load 6
**Performance measures**								
Incongruent RT (ms)	647.51 (130.39)	640.04 (135.15)	662.25 (132.19)	676.39 (140.44)	655.84 (134.90)	669.82 (137.20)	682.05 (126.06)	684.85 (155.54)
Congruent RT (ms)	612.59 (119.17)	622.57 (120.75)	623.03 (123.34)	636.90 (136.41)	643.13 (136.28)	634.36 (121.65)	637.68 (124.58)	651.06 (136.87)
WM error rate (%)	–	5.74 (0.23)	6.02 (0.24)	11.41 (0.32)	–	5.81 (0.23)	3.02 (0.17)	6.04 (0.24)
**Cardiac measures**								
BL HRV [ln(ms2)]	6.70 (1.10)	6.70 (1.07)	6.63 (1.11)	6.66 (1.02)	6.67 (1.09)	6.70 (1.09)	6.64 (1.11)	6.66 (1.02)
HRV ln(ms2)]	6.47 (1.14)	6.59 (1.19)	6.45 (1.16)	6.37 (1.17)	6.50 (1.19)	6.61 (1.15)	6.53 (1.17)	6.51 (1.23)
BL IBI (ms)	837.19 (123.32)	835.98 (131.64)	829.47 (124.52)	836.78 (131.07)	835.38 (123.29)	835.65 (130.80)	830.23 (124.00)	836.96 (131.52)
IBI (ms)	834.45 (120.61)	834.21 (130.53)	822.99 (125.71)	821.63 (129.21)	838.47 (125.30)	836.50 (123.82)	831.65 (125.88)	828.68 (129.86)
**Self-report**								
Task anxiety (Likert)	3.60 (1.69)	3.70 (1.61)	3.74 (1.73)	3.89 (1.61)	1.82 (1.08)	1.98 (1.12)	2.20 (1.19)	2.44 (1.27)
Trait Anxiety	38.01 (8.03)	Min = 21, Max = 61						

*RT, response time; WM, working memory; BL, baseline; HRV, heart rate variability; IBI, interbeat interval.*

#### Anxiety Manipulation on Cardiac Variables

Paired sample *t*-tests were conducted to examine HRV changes from baseline to threat and from baseline to safety. Compared to baseline, HRV was lower during threat, *t*(115) = 4.96, *p* < 0.001, Cohen’s *d*= 0.201, and safe trials, *t*(115) = 3.31, *p* = 0.001, Cohen’s *d*= 0.127. Baseline-to-task changes in IBI were handled with the same statistical approach. IBI contrasts for threat, *t*(115) = 1.95, *p* = 0.054, Cohen’s *d*= 0.058, and safety, *t*(115) = 0.328, *p* = 0.748, Cohen’s *d*= 0.008, were not significant. For a direct examination of threat-of-noise on HRV, a random intercept model containing Trial as a fixed effect was conducted on HRV during no load (i.e., 0 Load) trials, and there was no significant effect for Trial (*B* = 0.056, *p* = 0.255).

#### WM Load and Self-Reported Anxiety

The model above that tested effects of Trial (Threat, Safe) on self-reported anxiety was used to investigate WM load’s effect on diminishing anxiety. In addition to the Trial effect (see above), there was a significant positive association between WM Load and self-reported anxiety (*B* = 0.015, *p* = 0.005) for threat trials. There was also a significant interaction between Trial and WM Load (*B* = 0.036, *p* < 0.001), such that the positive association between WM Load and anxiety was stronger in safe relative to threat trials.

#### Anxiety Manipulation on Inhibition Performance

To substantiate that threat of noise blast negatively impacted inhibition performance, a multilevel model was conducted only on trials from 0 Load blocks. In this analysis, performance was the outcome measure and Trial was treated as a fixed effect. Inhibition performance was lower during unregulated threat compared to safe trials, as indicated by a significant effect of Trial (*B* = 22.39, *p* = 0.002).

### Primary Results

#### Hypothesis 1: WM Load and Inhibition Performance

The random intercept model examining Load effects on inhibition between threat and safety yielded significant effects for Trial (*B* = -13.83, *p* = 0.031) and WM Load^2^ (*B* = -1.31, *p* = 0.045). The main effect of WM Load^2^ was qualified by a significant WM Load^2^× Trial interaction (*B* = 3.36, *p* < 0.001). This interaction confirms that the quadratic relation between WM load and inhibition differs between threat and safe trials and justifies follow-up tests of WM Load effects for threat separately. See **Table 2** for a summary of random intercept models that tested load-inhibition relations.

**Table 2:** Multilevel Models: Fixed Effects of WM Load and WM Load^2^ on Inhibition and Task HRV.

	*A) Overall*				*B) Threat trials*				*C) Safety trials*				
	*B*	*SE*	*t*	σ ^2^	*B*	*SE*	*t*	σ ^2^	*B*	*SE*	*t*	σ ^2^
**Dependent measure: Inhibition performance (congruent minus incongruent)**												
Intercept	-28.05	4.94	-5.67ˆ**	617.95ˆ**	-27.38	4.82	-5.68ˆ**	788.81ˆ**	-44.00	5.17	8.52ˆ**	473.80ˆ**
Load	-1.62	1.17	-1.38		-1.52	1.14	-1.34		-3.26	1.25	-2.61ˆ**
Load^2^	-1.31	0.654	-2.01ˆ*		-1.31	0.631	-2.07ˆ*		2.13	0.667	3.12ˆ**
S-R anxiety	2.35	4.57	0.514		-6.24	7.99	-0.781		-3.88	7.11	-0.546
Trial	-13.83	6.41	-2.16ˆ*		-	-	-		-	-	-
Load X Trial	-1.94	1.66	-1.17		-	-	-		-	-	-
Load^2^ X Trial	3.36	0.919	3.66ˆ**		-	-	-		-	-	-
**Dependent measure: Task HRV (natural log of ms^2^)**												
Intercept	-0.082	0.048	1.67	0.112ˆ**	-0.119	0.048	-2.48ˆ**	0.124ˆ*	-0.074	0.046	-1.62	0.101ˆ**
Load	-0.006	0.010	-0.682		-0.008	0.010	-0.868		-0.005	0.010	-0.447
Load^2^	-0.015	0.005	2.76ˆ**		-0.014	0.005	-2.65ˆ**		-0.009	0.005	-1.70
S-R anxiety	-0.096	0.037	2.58ˆ*		-0.0006	-0.067	-0.009		0.001	0.066	0.019
Trial	-0.024	0.053	0.451		-	-	-		-	-	-
Load X Trial	0.007	0.014	0.519		-	-	-		-	-	-
Load^2^ X Trial	0.006	0.008	0.832		-	-	-		-	-	-

*Unstandardized regression coefficients are presented. *P*-value of fixed effects are for *t*-tests of slopes against zero. *P*-value of random effect are for Wald-*z* test of between-subject variance against zero. S-R, self-reported; HRV, heart rate variability. ^∗∗^p < 0.01, ^∗^*p* < 0.05.*

##### Quadratic relation between WM Load and inhibition under high state anxiety

**FIGURE 3:**
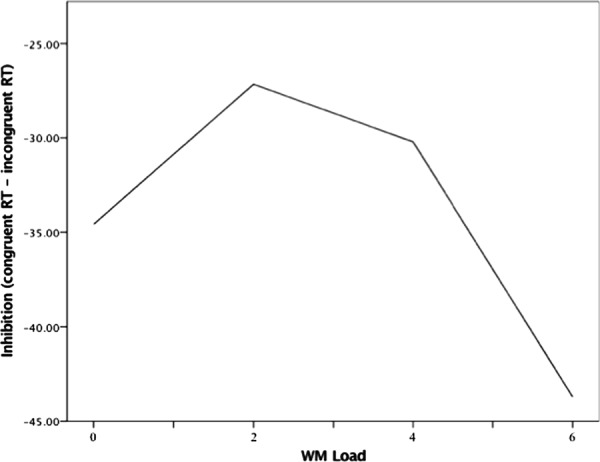
**Quadratic association between WM load and inhibition during threat of noise blast**. WM load levels: 0 (no load), 2 (low load), 4 (moderate load), and 6 (high load).

For the model that examined threat trials, only the effect of WM Load^2^ was significant (*B* = -1.31, *p* = 0.038), which indicated a negative quadratic function between WM load and inhibition performance under high state anxiety (i.e., threat). The precise shape of this function can be seen in **Figure 3**.

This quadratic relation can be explained as follows. Load increases from no to low load (0 to 2 digits) were associated with augmentations in performance, such that there was a positive load-inhibition relation. This positive relation reversed completely at low load (2 digits), whereby load increases from low to moderate load (2–4 digits) were met with decreases in inhibition performance (i.e., a negative relation). The negative relation grew stronger as load increased to 6 digits. The quadratic trend indicates that inhibition performance under anxiety is relatively better during low load (2 digits) compared to both no load and higher load (4 and 6 digits).

##### Negative linear relation between WM Load and inhibition under low state anxiety

**FIGURE 4:**
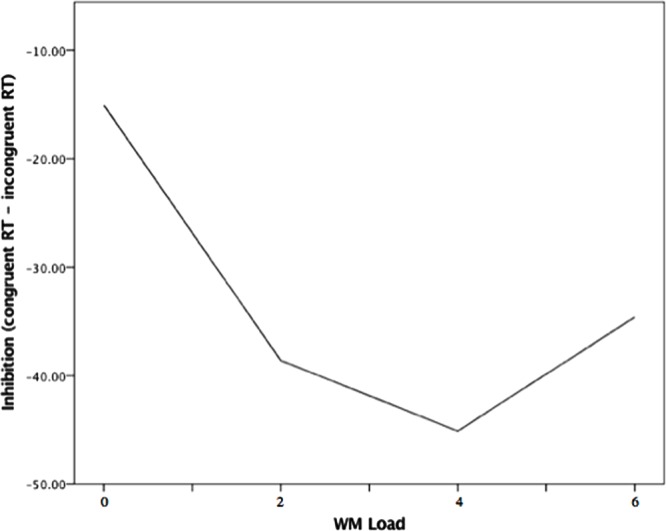
**Quadratic association between WM Load and inhibition during safety from noise blast.** Note. WM load levels: 0 (no load), 2 (low load),4 (moderate load), and 6 (high load).

The multilevel model examining load effects in safe trials indicated that there was a significant linear relation between WM Load and inhibition (*B* = -3.26, *p* = 0.009), but this linear effect was qualified by a significant quadratic association between WM Load and inhibition (*B* = 2.13, *p* = 0.001). As is seen in **Figure 4**, the negative relation appeared to attenuate and flatten across levels of load, until there was a slight reversal of the load-inhibition association from moderate to high load (4–6 digits).

#### Hypothesis 2: Task HRV and Load

**FIGURE 5:**
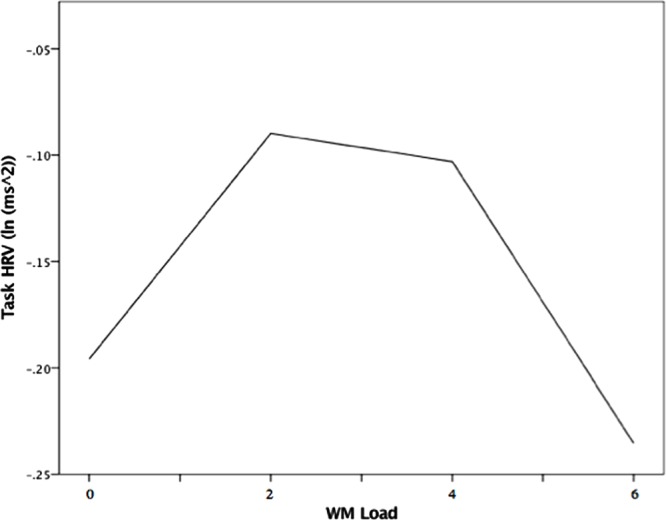
**Quadratic association between WM Load and Task IIRV during threat of noise blast, Note.** WM load levels: 0 (no load), 2 (low load),4 (moderate load), and 6 (high load).

The random intercept model that examined load effects on HRV between threat and safety revealed no significant interaction between WM Load and Trial (*B* = 0.007, *p* = 0.603). In examining threat trials singularly, the linear load-HRV relation was not significant (see model statistics in **Table 3**). However, for threat trials, there was an unpredicted significant effect for WM Load^2^ (*B* = -0.015, *p* = 0.006), as well as for Self-reported Anxiety (*B* = -0.096, *p* = 0.010). There were no significant effects in the model examining safety trials. Further inspection of WM Load’s quadratic effect under threat (see **Figure 5**) indicates that there was a positive load-HRV association from no to low load, which began to reverse from low to moderate low. The association then becomes increasingly negative, such that further increases in load past moderate levels (4 digits) were met with reductions in Task HRV.

**Table 3:** Multilevel models: fixed effects of task HRV and task HRV^2^ on inhibition.

	*A) Overall*				*B) Threat trials*				*C) Safety trials*				
	*B*	*SE*	*t*	σ ^2^	*B*	*SE*	*t*	σ ^2^	*B*	*SE*	*t*	σ ^2^
**Dependent measure: Inhibition performance (congruent minus incongruent)**											
Intercept	-31.88	4.27	-7.47ˆ**	614.33ˆ**	-32.87	4.15	-7.93ˆ**	773.11ˆ**	-28.98	3.92	-7.39ˆ**	465.06ˆ**
HRV	-1.87	3.71	-0.504		-1.21	3.63	-0.336		1.71	3.76	0.456
HRV^2^	-1.26	3.46	-0.364		-0.544	3.58	-0.152		-7.17	3.55	-2.02ˆ*
S-R anxiety	-1.99	4.56	-0.436		-9.14	8.08	-1.13		-10.13	7.93	-1.28
Trial	1.31	5.29	0.248		–	–	–		–	–	–
HRV X Trial	3.37	5.19	0.650		–	–	–		–	–	–
HRV^2^ X Trial	-3.91	4.66	-0.840		–	–	–		–	–	–

*Unstandardized regression coefficients are presented. P-value of fixed effects are for *t*-tests of slopes against zero. P-value of random effect are for Wald-*z* test of between-subject variance against zero. S-R, self-reported; HRV, heart rate variability. ^∗∗^*p* < 0.01; ^∗^*p* < 0.05.*

#### Hypothesis 3: Task HRV and Inhibition Performance

##### Quadratic relation between Task HRV and inhibition under high state anxiety

The multilevel model examining effects of HRV and HRV^2^ on inhibition between threat and safety indicated that there was no significant interaction between HRV^2^ and Trial (*B* = 3.91, *p* = 0.401). In fact, there were no significant main effects or interaction in this model. See **Table 3** for a summary of random intercept models examining HRV’s relations to inhibition. In the model examining threat trials only, the quadratic association between Task HRV and inhibition was not significant (*B* = 3.91, *p* = 0.401). In general, these data indicate that there were no associations between Task HRV and inhibition performance.

## Discussion

The primary aim of this study was to test for a negative quadratic relation between WM load and inhibition of distractors under high state anxiety, and to examine whether cardiac vagal control reflects WM load that both enhances and impairs inhibition under anxiety. Results partially confirmed hypotheses by showing a negative quadratic function between WM load and inhibition under high state anxiety. A number of unpredicted but potentially fruitful results emerged, which include a quadratic association between WM load and task HRV, in which HRV was highest under low load relative to all other load levels.

Contrary to hypotheses, there were no direct relations of HRV to inhibition. Findings suggest that under high state anxiety, the relation of WM load to distractor inhibition and cardiac vagal control depend on the availability of WM capacity (Lavie et al., 2004; Schmeichel et al., 2008; Thayer and Lane, 2009). As is discussed below, such availability, which might be reflected in task HRV, is the result of opposing effects of load-dependent anxiety reduction and load-dependent consumption of cognitive resources (Pessoa, 2009).

### Manipulation Checks and Model Tenets

Differences in anxiety ratings between threat and safety trials indicate that the noise blast paradigm was effective in inducing anxious cognition, as has been shown previously (Grillon and Ameli, 1998; Skolnick and Davidson, 2002; Lissek et al., 2005; Grillon et al., 2008). Further supporting the model and prior research, induced anxiety impaired inhibition, as is shown by worse inhibition in threat relative to safe trials during unregulated anxiety (e.g., Bishop et al., 2004; Hart et al., 2010; Choi et al., 2012). Contrary to the model and previous studies (e.g., van Dillen et al., 2009; Vytal et al., 2012; Patel et al., 2015), WM load increases were related to augmentations, and not reductions, in self-reported anxiety. It is possible that load reduced anxiety’s cognitive components (i.e., worry), which are related to WM, while bodily aspects of anxiety persisted (i.e., “anxious arousal) to be reflected in the self-report (Endler and Kocovski, 2001; Vytal et al., 2012; Sharp et al., 2015). Previous studies suggest that anxiety-related interoceptive cues can be detected consciously and thus self-reported; however, these interoceptive functions implicate neural functions that are not directly tied to WM (Nitschke et al., 1999; Critchley et al., 2004).

### Primary Findings

#### High State Anxiety: WM Load^2^ and Inhibition

Hypothesis 1 was partially supported in that there was a negative quadratic relation under high but not low state anxiety. Potential mechanisms that drive the non-linearity in the function can be clarified by focusing on its linear components, as is done below.

##### Inhibition enhancements

As hypothesized, there was a positive relation between load and inhibition in the range of no to low WM load. This positive relation is consistent with other studies in which relatively high load enhanced concurrent performance through attenuating negative emotional processing (Vytal et al., 2012; Clarke and Johnstone, 2013; Patel et al., 2015). High relative to low levels of WM load can speed reaction times to classify a happy target face amidst an angry distractor face, as well as reduce neural processing of the angry distractor face (van Dillen and Derks, 2012). As suggested in previous research, load increases in the present study may have enhanced inhibition by leaving less WM capacity for maintaining performance-harming anxiety (van Dillen and Koole, 2007). Prior studies have shown better performance under high versus low load, but the current study only showed a performance enhancement for low compared to no load, potentially because high load in the current task was especially demanding (three task demands) compared to prior research. Therefore, it is possible that present load effects across the entire function are restricted to a high range of WM load that is well beyond that of prior studies. That is, all of the present study’s load conditions might correspond to “high” load in other studies. Such a possibility is speculative, as a direct comparison of load conditions is difficult due to different tasks being used between studies (e.g., n-back, arithmetic problems, Sternberg WM task). Yet, lack of inhibition enhancements from no to low load under safety supports the notion that enhancements during threat were caused by anxiety reductions, because safety did not likely involve enough anxiety to allow for notable load-dependent anxiety reductions.

##### Inhibition impairments

In accord with the model, there was a reversal of the positive linear relation between load and inhibition under anxiety such that the relation became negative from low to moderate load. As WM capacity became increasingly scarce, higher load related to relatively worse inhibition under anxiety. The negative load-inhibition relation became even stronger from moderate to high levels of load. These findings may be due to reliance of distractor inhibition on limited WM capacity (Baddeley, 1992; Engle, 2002), and because high load tends to worsen inhibition of irrelevant visual distractors (Lavie, 2005, 2010). The reversal and intensification of the load-inhibition relation suggests that competition between WM load and other cognitive functions (e.g., inhibition) may be stronger when WM capacity limits are reduced and resources are scarce (Cowan, 2001; Pessoa, 2009; Forster, 2013). In effect, as WM was increasingly depleted past low load, performance may have been impaired in proportion to capacity availability, such that load-induced impairments increasingly outweighed concurrent load-dependent performance improvements. As mentioned above, it is possible that load’s impairments to inhibitions require heavy taxation of WM capacity. Previous studies may have missed this section of the function because the present study’s added task demands made the 6-digit condition sufficiently high to impair inhibition (e.g., van Dillen and Derks, 2012; Vytal et al., 2012).

##### Revising the Theoretical Model

A discrepancy between the yielded function and the model (**Figure 1**) is that there was neither attenuation nor a plateau in the positive relation at moderate load. In effect, inhibition performance under anxiety was optimal under low rather than moderate load. It is possible that performance would have been even better if three digits were maintained, a condition not included in this study. Another possibility is that low load represents a meaningful level of WM usage past which further load increases drain resources needed for inhibition. If the latter is the case, a logical query arises as to why load increases from no to low load were uniquely associated with inhibition enhancements rather than impairments. A potential explanation might relate to the fact that: (1) emotion-related cognition demands more cognitive resources than low load neutral cognition (Vytal et al., 2012), and (2) the attenuation of anxiety by load is stronger under high relative to low anxiety (van Dillen and Koole, 2007; Stout et al., 2013). By shifting resources away from heavily depleting anxiety, low load likely frees up much more WM capacity than it fills with digit maintenance alone, and this effect may improve concurrent inhibition performance.

Compared to low load neutral information, threatening stimuli strongly consume WM capacity, as measured by neural and behavioral measures (Dolcos and McCarthy, 2006; Stout et al., 2013). In the current study, poor inhibition at no relative to low load may have been caused by unregulated anxiety (0 Load) draining more shared resources than low load maintenance (e.g., Hajcak and Olvet, 2008; Kanske et al., 2011; Dolcos and Denkova, 2014). Second, smaller amounts of WM load might be more effective at clearing anxious cognition from WM capacity when WM resources are increasingly used by these cognitions (van Dillen and Koole, 2007). Thus, from no to low load (when there is increased anxious cognition in WM capacity), minimal task-related increases in load may have dissipated anxious cognition and thus had a net effect of freeing up more WM capacity than was filled by low load manipulation (i.e., 2 digits). As such, this free capacity was available for the concurrent inhibition task. With further increases in task-related load, however, inhibition may have declined because less WM capacity was free for maintaining inhibition goals (Miller and Cohen, 2001; Lavie et al., 2004; Qi et al., 2014). This “capacity availability” account of results is supported by unexpected HRV findings, as is discussed below.

#### Quadratic Relation between Load and Task HRV

It was hypothesized that task HRV would reflect WM load used to decrease anxious cognition. Rather than supporting a linear relation between HRV and WM-dependent regulation over anxiety, a more complex non-linear association between WM load and HRV was observed. This quadratic association aligns with elements of the Neurovisceral Integration Model and past studies that view vagal control not as an index of degree of cognitive regulation, but as a reflection of PFC resources available for ongoing cognitive-affective demands (Jorna, 1992; Elliot et al., 2011). In fact, Thayer and Lane (2009, p. 85) have suggested that “HRV functions at both the trait and state levels as a resource.”

Heart rate variability changes in response to load followed a similar trend to that of load-induced performance changes. Task HRV was highest at low levels of load (2 digits), when inhibition was optimized; then, the load-HRV association reversed from low to moderate load just as the load-inhibition association did (see **Figures 3** and **5**). Since inhibition is dependent on WM availability, it is possible that task HRV reflected the degree of “free” WM capacity that resulted from both anxiety reduction and load itself (Engle, 2002; Lavie, 2010). At low load, when inhibition performance was optimized, HRV may have reflected a large amount of WM resources that were salvaged through “deleting” anxious cognition and made available for removal of distractor interference (Dolcos and Denkova, 2014). The decline of HRV after low load may reflect WM capacity being increasingly filled with task-related load, consistent with the parallel load-dependent decreases in performance seen in **Figure 3** (Croizet et al., 2004). If HRV is interpreted as an indicator of resource availability, the yielded load-HRV quadratic relation is consistent with studies in which task HRV was negatively related to ongoing task demands and positively related to cognitive performance that requires high levels of available WM capacity (Hansen et al., 2003; Lehrer et al., 2010; Elliot et al., 2011; Allen and Friedman, 2016).

The absence of the predicted quadratic association between HRV and inhibition conflicts with our finding of a quadratic function between HRV and executive function in those who frequently use a WM-dependent ER strategy (Spangler et al., 2015). This quadratic association included resting HRV, which unlike phasic HRV, has been theoretically linked to trait processes whereby ER’s costly effects potentially accrue over time (Butler et al., 2006). Although there has been one report of a quadratic association between task HRV and executive function in children (Marcovitch et al., 2010), it might be that task HRV taps into the state-related availability of resources that can be used for inhibition of distraction.

### Implications for ER and Intervention

Since many ER strategies entail WM loading, the present findings qualify theoretical perspectives in which ER is held to assist performance via the use of executive control (Thayer and Lane, 2000; Blair and Ursache, 2011; Cohen et al., 2012). Cognitive regulation of high anxiety may only enhance concurrent inhibition insofar as that regulation does not heavily load WM. Clarification is also given to the view of ER as damaging to attentional focus by suggesting that ER strategies may only hurt performance when they are highly loading, as in the case of expressive suppression (Kalisch et al., 2006; Goldin et al., 2008; Friese et al., 2013; Ortner et al., 2013).

Regarding cardiac vagal control, our findings indicate that deploying WM resources in the service of ER does not cause simple increases in HRV, as might be predicted from previously shown HRV augmentations during ER. The present results instead suggest that on-task HRV levels reflect inter-function competition of WM-related regulation, anxiety, and inhibition. This view is somewhat inconsistent with the Neurovisceral Integration Model, which highlights the anatomical-functional integration of ER and “cold” executive functions, which work together in self-regulation (Thayer and Lane, 2009). However, by virtue of integrated neurocognitive resources in the PFC, there is inherent resource competition between emotion, ER, and executive control, of which HRV might be a reflection (Pessoa, 2008, 2009).

The current study also underscores the potential value of using minimally loading ER strategies for treatment in anxiety disorders, of which a major feature is difficulty in concentration (Beck et al., 2005). Interventions like CBT that involve complex cognitive ER strategies (e.g., reappraisal) may do more harm than good by impairing anxious individuals’ ability to inhibit irrelevant information, and in doing so, worsen anxious symptoms (Olatunji et al., 2007). ER strategies might be better chosen according to their level of load, so that damaging effects on attention and daily functioning are minimized.

### Limitations, Future Directions, and Concluding Remarks

The present study has limitations that might be addressed in future research on the relationships among load, inhibition, and HRV under anxiety. First, state anxiety was only measured via self-report, which has been shown to diverge from other aspects of anxiety (Sharp et al., 2015). Future studies might include measures of eyeblink startle to more comprehensively assess anxious states and to better substantiate the left side of the yielded non-linear functions (Grillon, 2008). There was also no direct WM capacity measure in this study. Future research could include neuroimaging to more directly index resource competition at the central nervous system level. Although HRV data from noise blast trials were removed from analyses, it is conceivable that the noise blasts influenced HRV estimates in surrounding trials. This possibility is somewhat unlikely, as cardiac vagal responses to noise blast return to baseline levels within a time period (i.e., three to four heartbeats; <5 s) shorter than the present study’s intervals between HRV measurements (Chen et al., 2014). This study also had a number of strengths that should be noted, including a relatively large sample size and many within-subjects observations. These factors allowed for a powerful test of hypothesized three-way interactions (e.g., quadratic effects varying between safety and threat).

In sum, this study provides evidence that minimal WM load can attenuate the impairing effects of anxiety on distractor inhibition, while more heavily loading tasks may do just as much harm to inhibition as anxiety itself. The current study also underscores cardiac vagal control as a potential correlate of WM resource availability, a factor that relates to attentional performance under threat. Broadly speaking, this study may inform treatments for anxiety disorders, in which regulation of emotion and anxiety can be modified to prevent lapses in attention.

## Ethics Statement

This study was approved by the Institutional Review Board (IRB) at Virginia Tech. All participants were greeted and the experimenter explained each section of the informed consent form, in order to educate participants on the nature of the study procedures and purpose, risks and benefits, as well as their freedom to withdraw at any point time with no penalty. Participants were then given the opportunity to ask questions, after which they signed the informed consent form. No vulnerable populations were used in this study.

## Author Contributions

DS wrote this under the mentorship and direction of BF. DS developed the research question and conducted this study as a part of his dissertation, with BF providing invaluable feedback and edits on the project’s implementation and on the submitted manuscript

## Conflict of Interest Statement

The authors declare that the research was conducted in the absence of any commercial or financial relationships that could be construed as a potential conflict of interest.
